# Retraction: Entrapment of H1N1 Influenza Virus Derived Conserved Peptides in PLGA Nanoparticles Enhances T Cell Response and Vaccine Efficacy in Pigs

**DOI:** 10.1371/journal.pone.0307483

**Published:** 2024-07-16

**Authors:** The PLOS ONE Editors

Following publication of this article [1], a concern was raised about the data reported in the charts in Fig 4. Underlying data were provided by corresponding author GJR, and editorial assessment of the data identified discrepancies compared to the published figure and a further concern about flow cytometry data in Fig 2A.

Following an institutional investigation, The Ohio State University notified the *PLOS ONE* Editors that the article contains falsified data and recommended retraction of the article [1]. Specifically the editors were notified of the following investigation findings:

- In Fig 2A of [1], a flow cytometry dot plot for CD4/CD8 cell gating from peripheral blood mononuclear cells (PBMCs) is used to represent a dot plot for CD4/CD8 cell gating from lung cells. The same dot plot is also published in Fig 5 of [2], in which it is reported to show CD4/CD8 gating of PBMCs from pigs treated with a different vaccine.
- Fig 4A in [1] contains falsified rectal temperature data; the reported day -1 values in Fig 4A are inconsistent with the raw data.

In light of the above concerns related to the validity and integrity of the reported results, the *PLOS ONE* Editors retract this article.

JH, KK, SD, CWL, and GJR agreed with the retraction. MX, ME, BB, KO, JA, JBT, and XJ either did not respond directly or could not be reached. SD, CWL, and GJR apologize for the issues with the published article.
